# Synthesis, CYP24A1-Dependent Metabolism and Antiproliferative Potential against Colorectal Cancer Cells of 1,25-Dihydroxyvitamin D_2_ Derivatives Modified at the Side Chain and the A-Ring

**DOI:** 10.3390/ijms21020642

**Published:** 2020-01-18

**Authors:** Magdalena Milczarek, Michał Chodyński, Anita Pietraszek, Martyna Stachowicz-Suhs, Kaori Yasuda, Toshiyuki Sakaki, Joanna Wietrzyk, Andrzej Kutner

**Affiliations:** 1 Hirszfeld Institute of Immunology and Experimental Therapy, Polish Academy of Sciences, 12 Rudolfa Weigla, 53-114 Wroclaw, Poland; martyna.stachowicz@hirszfeld.pl (M.S.-S.); joanna.wietrzyk@hirszfeld.pl (J.W.); 2 Department of Chemistry, Pharmaceutical Research Institute, 8 Rydygiera, 01-793 Warsaw, Poland; m.chodynski@ifarm.eu (M.C.); a.pietraszek@ifarm.eu (A.P.); 3 Department of Pharmaceutical Engineering, Toyama Prefectural University, Toyama 939-0398, Japan; kyasuda@pu-toyama.ac.jp (K.Y.); tsakaki@pu-toyama.ac.jp (T.S.); 4 Department of Bioanalysis and Drug Analysis, Faculty of Pharmacy with the Laboratory Medicine Division, Medical University of Warsaw, 1 Banacha, 02-097 Warsaw, Poland; akutner@chem.uw.edu.pl

**Keywords:** vitamin D derivatives, double-point modified vitamin D_2_ analogs, 1,25-dihydroxyergocalciferol analogs, CYP24A1-dependent metabolism, antiproliferative activity *in vitro*, human colorectal cancer cell lines

## Abstract

Experimental data indicate that low-calcemic vitamin D derivatives (VDDs) exhibit anticancer properties, both *in vitro* and *in vivo*. In our search for a vitamin D analog as potential anticancer agent, we investigated the influence of chirality in the side chain of the derivatives of 1,25-dihydroxyergocalciferol (1,25D2) on their activities. In this study, we synthesized modified analogs at the side chain and the A-ring, which differed from one another in their absolute configuration at C-24, namely (24*S*)- and (24*R*)-1,25-dihydroxy-19-*nor*-20a-homo-ergocalciferols (PRI-5105 and PRI-5106, respectively), and evaluated their activity. Unexpectedly, despite introducing double-point modifications, both analogs served as very good substrates for the vitamin D-hydroxylating enzyme. Irrespective of their absolute C-24 configuration, PRI-5105 and PRI-5106 showed relatively low resistance to CYP24A1-dependent metabolic deactivation. Additionally, both VDDs revealed a similar antiproliferative activity against HT-29 colorectal cancer cells which was higher than that of 1,25D3, the major biologically active metabolite of vitamin D. Furthermore, PRI-5105 and PRI-5106 significantly enhanced the cell growth-inhibitory activity of 5-fluorouracil on HT-29 cell line. In conclusion, although the two derivatives showed a relatively high anticancer potential, they exhibited undesired high metabolic conversion.

## 1. Introduction

Currently, there are numerous vitamin D derivatives (VDDs) at various stages of development for clinical applications in the treatment of hyperproliferative diseases including leukemias and solid tumors [1,2,3]. A low level of calcemic activity, compared to the parent vitamin D hormone, high affinity to vitamin D receptor (VDR) and high resistance to CYP24A1-dependent catabolism are the prerequisites to be met by the vitamin D analogs to qualify for clinical trials [4,5]. The other important factors that are evaluated at the pre-clinical level include high affinity to serum vitamin D-binding protein, which is linked with pharmacokinetics, selective interaction of VDR with co-activators (SRC-1, TIF2, AIB-1), tissue distribution and catabolism by enzymes other than CYP24A1 [6].

In our previous study, the 1,25-dihydroxyergocalciferol (1,25-dihydroxyvitamin D_2_, 1,25D2) analogs synthesized by us, PRI-5105 and PRI-5106, showed a relatively high antiproliferative and prodifferentiating activity on human HL-60 and MV4-11 leukemic cell lines, combined with a low calcemic activity *in vivo*, and proved as promising candidates for further preclinical development [7]. Subsequently, we developed a method for practical and efficient convergent synthesis of PRI-5106 and its 24-diastereomer, PRI-5105, starting from three known building blocks. In this study we have complemented the previously published biological profiles of these analogs [7], analyzing their anticancer potential on colorectal cancer (CRC) cell lines as well as their CYP24A1-dependent metabolism, compared to 1,25-dihydroxycholecalciferol (1,25-dihydroxyvitamin D_3_, 1,25D3) as a gold standard. The binding affinities of these analogs to VDR were already investigated in another study [8]. The results showed that the absolute configuration at C-24 in the 19-*nor* analogs, which have a vitamin D_2_-like side chain that is extended by one carbon (C-20a), influences the VDR affinity. Compared to 1,25D3, the affinity of PRI-5105 was significantly reduced while that of PRI-5106, its diastereomer, was slightly augmented. However, the decrease of VDR-binding affinity [8] did not affect the potency of PRI-5105 to induce differentiation of HL-60 cells in comparison to 1,25D3 [7,8].

In our previous *in vivo* studies on a murine MC38 and human HT-29 CRC model, we showed that 1,25D3 analogs, especially tacalcitol [(24*R*)-1,24-dihydroxyvitamin D_3_, PRI-2191], significantly enhance the anticancer activity of 5-fluorouracil (5-FU) [9,10]. In this regard, we performed this study to evaluate the effect of 1,25D2 analogs, PRI-5105 and PRI-5106, alone and along with 5-FU, on the proliferation of human HT-29 and HCT116 CRC cell lines. These cell lines were selected since they differ in the differentiation stage and belong to different consensus molecular subtypes (CMS) of CRC [11]. HT-29 cells belong to CMS3 and are classified as a subgroup of colon-like cell lines expressing gastro-intestinal differentiation markers, while HCT116 cells belong to CMS4 and are classified as undifferentiated cell lines showing upregulated epithelial-mesenchymal transition (EMT) and TGFβ signatures. Additionally, HCT116 cells show genomic instability phenotypes and are microsatellite unstable, whereas HT-29 cells are microsatellite stable [11]. However, both these cell types represent right-side colon cancer [12].

## 2. Results and Discussion

### 2.1. Synthesis and CYP24A1-Dependent Metabolism

**Figure 1:**
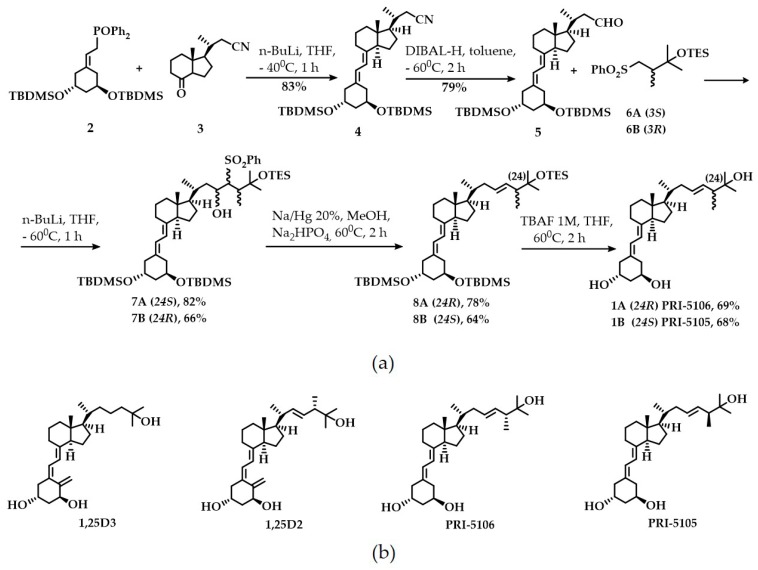
Synthesis and structures of the vitamin D derivatives (VDDs) studied. (**a**) Synthesis of VDDs of 1,25D2: PRI-5106 (**1A**) and PRI-5105 (**1B**); (**b**) Structures of 1,25-dihydroxyvitamin D_3_ (1,25D3) and 1,25-dihydroxyvitamin D_2_ (1,25D2) as well as (24*R*)-1,25-dihydroxy-19-*nor*-20a-homo-ergocalciferol (PRI-5106) and (24*S*)-1,25-dihydroxy-19-*nor*-20a-homo-ergocalciferol (PRI-5105).

In our convergent synthesis (Figure 1a), both the target VDDs, PRI-5105 and PRI-5106, were constructed from three structural fragments: the A-ring fragment **2**, the CD ring system **3**, and the side-chain fragment **6A** or **6B**. Wittig-Horner coupling of the known A-ring phosphine oxide **2** with the CD-ring C-22 nitrile **3** [13] gave rise to the key ACD synthon **4** with a yield of 83%. Nitrile **4** was hydrolyzed to obtain the respective C-23 aldehyde **5** with 79% yield. Julia olefination of aldehyde **5** with the side-chain chiral (3*S*) sulfone **6A** led to the mixture of (24*S*) diastereomeric hydroxysulfones **7A** with a yield of 82%. Radical dehydroxy-desulfonylation of **7A**, followed by fluoride-promoted simultaneous three-centered desilylation at C-1, C-3 and C-25, resulted in the final (24*R*) analog **1A** (PRI-5106) with an overall yield of 28.9% (starting from **3**). (24*S*) analog **1B** (PRI-5105) was synthesized the same way using (3*R*) sulfone **6B** with an overall yield of 18.8% (starting from **3**). Before performing analytical determinations and biological evaluation both **1A** and **1B** analogs were purified by reversed-phase high-performance liquid chromatography (HPLC) followed by crystallization to achieve compounds that can show a single peak on the HPLC chromatograms.

We analyzed the metabolism of VDDs, PRI-5105 and PRI-5106, by recombinant human CYP24A1 (*h*CYP24A1) as described in the “Materials and Methods” section. The HPLC profiles of the VDDs and their metabolites are shown in Figure S1 in the Supplementary Materials. The metabolic conversion of PRI-5105 and its diastereomer, PRI-5106, by *h*CYP24A1 was estimated as 25 and 26%, respectively, while that of the active metabolite of vitamin D_2_, 1,25D2, as 33% (Table 1).

In our previous studies [14], both the active metabolites of vitamin D, 1,25D3 and 1,25D2 (Figure 1b), showed high CYP24A1-dependent metabolism, as 44 and 35%, respectively. Compared to our recently studied analogs of vitamin D_2_ with side-chain modifications (PRI-1906 and PRI-1907) as well as their 19-*nor*-modified derivatives (PRI-5201 and PRI-5202, respectively) (Figure S2), which showed a metabolic conversion value of 2.3, 0.9, 1.9 and 2.0%, respectively [14,15], the side-chain-modified 19-*nor* analogs of 1,25D_2_, PRI-5105 and PRI-5106, reported in the present study showed very high degradation. The VDDs PRI-5105 and PRI-5106, which differ only in their chirality at C-24, showed a similar level of metabolic conversion, indicating that resistance to CYP24A1-dependent metabolism is not highly dependent on their absolute configuration at this chiral center. Similarly, the 19-*nor* modification of both these VDDs did not substantially increase their resistance to CYP24A1-dependent metabolism. Additionally, compared to their parent 1,25D2, the side chain of both PRI-5105 and PRI-5106 was extended by a single carbon unit, similar to the side chain of the VDDs PRI-5201 and PRI-5202. Thus, the only structural difference that influenced the metabolic conversion of the analogs was in the structure of the terminus of the side chain. PRI-5105 and PRI-5106 contained an extended vitamin D_2_-like side chain, while PRI-5201 and PRI-5202 were characterized by conjugated unsaturation in their extended side chain.

### 2.2. Anticancer Potential

**Figure 2:**
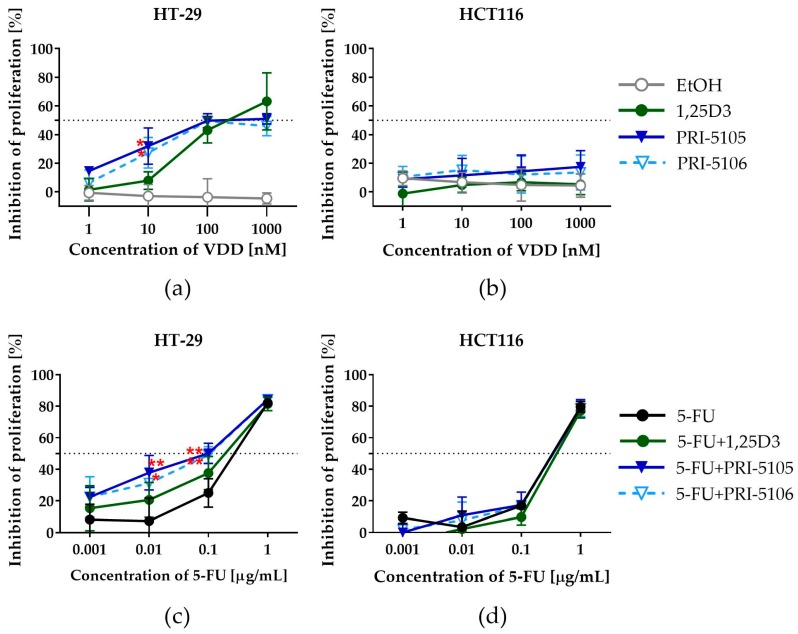
Antiproliferative activity of PRI-5105 and PRI-5106 alone and in combination with 5-FU toward HT-29 and HCT116 CRC cell lines. (**a**,**b**) The percentage values of the proliferation inhibition of cells exposed to VDDs used at the concentrations of 1, 10, 100 and 1000 nM. (**c**,**d**) The percentage values of the proliferation inhibition of cells exposed to VDDs at the concentrations of 10 nM combined with 5-fluorouracil (5-FU) applied at the concentrations of 0.001, 0.01, 0.1 and 1 µg/mL. The cells were incubated with the compounds for 120 h (5 days). Data represent the mean ± SD of four independent experiments. Statistical analysis was carried out using parametric one-way analysis of variance (ANOVA) followed by Dunnett’s multiple comparisons test (* *p* ≤ 0.05, and ** *p* ≤ 0.01 as compared to 1,25D3 (**a**) and 5-FU (**b**)).

PRI-5105 and PRI-5106 were also evaluated for their antiproliferative activity *in vitro* against human HT-29 and HCT116 CRC cell lines as compared to 1,25D3, the major biologically active metabolite of vitamin D. It was observed that the antiproliferative activity of both VDDs toward HT-29 CRC cells was at a similar level, but the compounds exhibited higher activity than 1,25D3 in a dose-dependent manner (Figure 2a). Significant differences in activity were observed at a low concentration (10 nM), whereas at the highest concentration used (1000 nM) the activity of all compounds was found to be similar. However, both analogs and 1,25D3 showed no significant differences in their activity toward HCT116 cells (Figure 2b). Moreover, the HT-29 CRC cell line revealed a higher sensitivity to the antiproliferative effect of VDDs than HCT116 cells (Figure 2a,b). We also investigated the influence of VDDs on the anticancer activity of 5-FU *in vitro* against the CRC cell lines (Figure 2c,d). Both PRI-5105 and PRI-5106 significantly enhanced the cell growth-inhibitory activity of 5-FU on HT-29 cells (Figure 2c) leading to a 2.9- or 2.7-fold decrease in its inhibitory concentration 50% (IC_50_) values (Table 2), respectively. Additionally, the combination of 5-FU with PRI-5105 or PRI-5106 used at a concentration of 10 nM caused synergism. On the other hand, 5-FU applied with 1,25D3 indicated additivity (Table 2). However, we did not observe any change in the anticancer activity of 5-FU toward HCT116 cells (Figure 2d) due to their poor sensitivity to the proliferation-inhibiting effect of the studied analogs.

A comparison with our previous studies showed that the antiproliferative activity of PRI-5105 and PRI-5106 toward HT-29 cells was similar to that of PRI-1906, PRI-1907, PRI-5201, PRI-5202 and 1,25D3 at the concentration of 1000 nM [7]. However, in this study, we observed that both PRI-5105 and PRI-5106 were more active against HT-29 cells than 1,25D3 at a lower concentration (10 nM). These data suggest that 1000 nM is too high concentration to notice the differences in the activity of VDDs. The literature data indicate that the analogs that reached the clinical trials have not demonstrated convincing anticancer effects, which may be due, at least in part, to their dose-limiting calcemic toxicity [4,14,16]. Therefore, researchers search for VDDs that are capable of sensitizing the cancer cells even at low concentrations [14,16]. In view of this, we investigated whether PRI-5105 and PRI-5016 also influence the anticancer activity of 5-FU against the CRC cells when used at a low concentration. We observed that both the studied VDDs sensitized HT-29 cells to the activity of 5-FU and contributed to the decrease of its IC_50_ values. Potential reduction of the doses of chemotherapeutics, achieved by adding VDDs, for clinical application could result in fewer side effects without any compromise on the efficacy of the treatment. However, this effect was observed only in the case of HT-29 cells which are more sensitive to VDDs and not in HCT116 cells.

**Figure 3:**
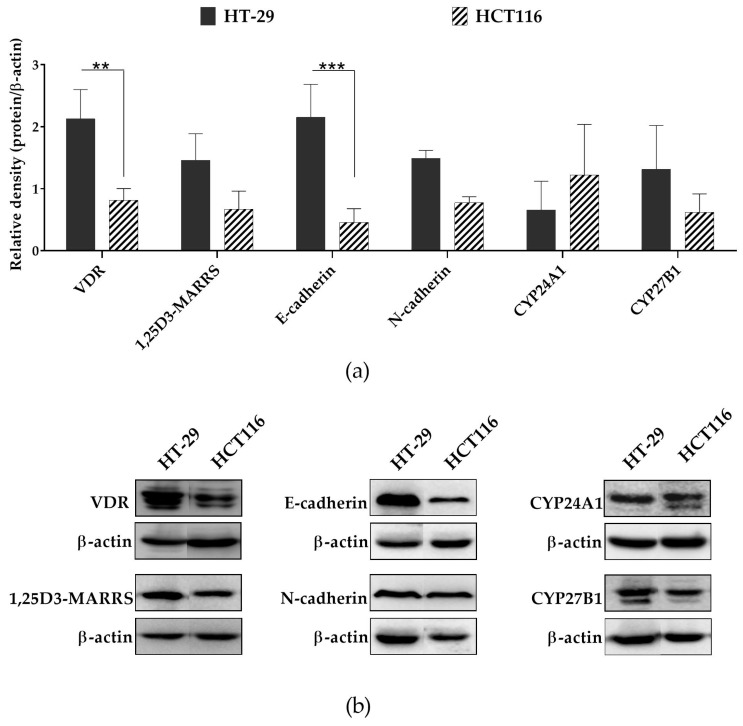
The basal expression of proteins involved in the metabolism and anticancer activity of 1,25D3 and its analogs in HT-29 and HCT116 cells evaluated by western blot. (**a**) Data represent the results obtained from the densitometric analysis of the bands of protein tested as a ratio to β-actin (mean ± SD of three independent experiments). Statistical analysis was carried out using parametric two-way ANOVA followed by Sidak’s multiple comparison test (** *p* ≤ 0.01, *** *p* ≤ 0.001). (**b**) Representative immunoblots of the proteins of interest.

Our previous results indicate that VDR might be useful as a molecular marker to predict treatment outcomes and identify the CRC patients who might benefit from the 5-FU-based chemotherapy combined with VDD [17]. However, the low level of VDR protein, which also participates in the action of 5-FU and may prevent the development of resistance to 5-FU treatment in CRC cells [17], could be the reason for treatment failure. The presence of VDR does not guarantee the response of the CRC cells to VDDs. This fact was evident in the case of HCT116 cells. Both HT-29 and HCT116 cell lines showed the expression of VDR at the protein level, but the former revealed a significantly higher level of VDR than the latter (Figure 3a,b). According to the COSMIC Cell Line Project database, *VDR* exists as a wild-type gene in both cell lines. However, these cell lines differ in their differentiation stage and belong to different CMS of CRC. HT-29 cells belong to the subgroup of colon-like cell lines, while HCT116 cells are classified as an undifferentiated cell line showing upregulated EMT [11]. During EMT, the expression of E-cadherin is downregulated while that of N-cadherin is upregulated [18]. Thus, we noticed an enhanced ratio of E-cadherin to N-cadherin in HT-29 cells compared to HCT116 cells (Figure S3). Additionally, HT-29 cell line revealed a significantly higher level of E-cadherin than HCT116 cells (Figure 3a). Furthermore, according to the COSMIC database, in HCT116 cells occurs mutation in *CDH1* gene (p.H121fs*94) (gene encoding E-cadherin), which might result in the formation of nonfunctional E-cadherin that leads to changes in the phenotype of cells from immobile epithelial to invasive mesenchymal ones and may consequently contribute to the development of resistance to VDDs. We also noticed that HCT116 cells showed a higher level of CYP24A1 than that of CYP27B1, while an inverse effect was observed in the case of HT-29 cells (but the difference was not significant) (Figure 3a,b, Figure S3). This might consequently result in the low concentration of active VDDs in HCT116 cells. Taken together, the differences in the response of CRC cell lines to VDDs depend on the molecular features of the cancer cells including their differentiation stage, the degree of expression of molecules that are important in the metabolism and anticancer action of VDDs, and the occurrence of the mutations in these molecules. Thus, understanding of the molecular factors underlying the differences in the response of CRC cells to VDDs is highly desirable, as it might allow identifying the CRC patients who could benefit from the 5-FU- and VDD-combined chemotherapy.

## 3. Materials and Methods

### 3.1. Synthetic and Analytical Chemistry

^1^H and ^13^C NMR spectra were recorded in deuterochloroform at ambient temperature using a Varian VNMRS-600 instrument. Chemical shift values are quoted in parts per million and J values in Hertz. IR spectra were recorded on a Perkin-Elmer 1600 series FT-IR spectrometer. Mass spectra were obtained on a Finnigan MAT Model 8200 spectrometer. UV spectra were taken on a Shimadzu Model 160A UV-VIS spectrophotometer in the solvents indicated. Column flash chromatography was performed on silica gel Si 60 (230–400 mesh, Merck, Darmstadt, Germany). Reactions were monitored by thin-layer chromatography (TLC) performed using Merck Kieselgel 60 F_254_ plates. The TLC plates were visualized with a combination of UV light and an aqueous solution of cerium (IV)/phosphomolybdenic acid at a high temperature. The analytical and preparative separations of HPLC were performed using a Knauer Model 64 instrument with Eurospher 100 C18 column (40 × 250 mm). All moisture-sensitive reactions were performed in flame-dried glassware under argon.

### 3.2. Synthesis

#### 3.2.1. (*1R*,*3R*,*7E*,*20R*)-1,3-*Bis*(*tert*-butyldimethylsilyloxy)-20-methyl-19-*nor*-9,10-secopregna-5,7-diene-21-carbonitrile **4**

To a solution of phosphine **2** (10.3 g, 18.1 mmol) in THF (25 mL), *n*-BuLi (1.6 M in hexane, 13.5 mL, 21.6 mmol) was added at 0 °C. The reaction mixture was cooled in a dry ice bath, and a solution of ketonitrile **3** (2.8 g, 12.8 mmol) in THF (10 mL) was added dropwise at −50 °C. The mixture was stirred for 60 min at −40 °C. After warming up to room temperature (RT), water was added and the product was extracted with ethyl acetate (3 × 50 mL). The organic layer was washed with brine and dried over MgSO_4_ (10 g). The filtrate was concentrated *in vacuo*. The residue was purified by silica gel chromatography to obtain the corresponding nitrile **4** (5.4 g, 83%) as a colorless oil. ^1^H-NMR (CDCl_3_) δ 0.05 (3H, s, Si-CH_3_), 0.06 (3H, s, Si-CH_3_), 0.56 (3H, s, 18-CH_3_), 0.86 (9H, s, Si-C(CH_3_)_3_), 0.88 (9H, s, Si-C(CH_3_)_3_), 1.18 (3H, d, *J* = 6.6 Hz, 21-CH_3_), 4.07 (2H, m, 1-H and 3-H), 5.81 (1H, d, *J* = 11.4 Hz, 7-H), 6.16 (1H, d, *J* = 11.4 Hz, 6-H); ^13^C-NMR (CDCl_3_) δ 12.1, 18.0, 18.1, 19.4, 22.1, 23.1, 24.7, 25.8, 26.9, 27.5, 28.5, 33.9, 36.8, 40.1, 43.6, 45.5, 45.9, 55.1, 55.9, 67.9, 68.0, 116.5, 118.9, 121.5, 134.1, 139.8; UV (C_2_H_5_OH) λ_max_ = 261.2 nm, λ_max_ = 251.8 nm, λ_max_ = 243.2 nm; IR (thin film) ν_max_ 2953, 2929, 2884, 2856, 1734, 1618, 1471, 1384, 1361, 1255, 1088, 1052, 1027, 836, 774 cm^−1^; MS *m*/*z* 571 (3, M^+^).

#### 3.2.2. (*1R*,*3R*,*7E*,*20R*)-1,3-*Bis*(*tert*-butyldimethylsilyloxy)-20-methyl-19-*nor*-9,10-secopregna-5,7-diene-21-carboaldehyde **5**

Nitrile **4** (5.4 g, 9.4 mmol) and toluene (100 mL) were added to a round-bottomed flask. The mixture was cooled in a dry ice bath, and a solution of DIBAL-H (1 M in toluene, 25 mL, 25 mmol) was added dropwise at −60 °C. After stirring for 90 min, the mixture was warmed up to RT and brine (50 mL) was added. The resulting product was extracted with ethyl acetate (4 × 50 mL). The combined organic layer was washed with water and dried over MgSO_4_ (20 g). The residue was purified by silica gel chromatography to obtain aldehyde **5** (4.3 g, 79%) as a colorless oil. ^1^H-NMR (CDCl_3_) δ 0.05 (3H, s, Si-CH_3_), 0.06 (3H, s, Si-CH_3_), 0.59 (3H, s, 18-CH_3_), 0.86 (9H, s, Si-C(CH_3_)_3_), 0.87 (9H, s, Si-C(CH_3_)_3_), 1.03 (3H, d, *J* = 6.6 Hz, 21-CH_3_), 4.08 (2H, m, 1-H and 3-H), 5.81 (1H, d, *J* = 11.0 Hz, 7-H), 6.16 (1H, d, *J* = 11.0 Hz, 6-H), 9.76 (1H, m, -CHO); ^13^C-NMR (CDCl_3_) δ 12.0, 18.2, 18.4, 19.5, 22.1, 23.1, 24.8, 25.7, 26.9, 27.5, 28.6, 33.7, 36.8, 40.2, 43.6, 45.5, 45.9, 55.5, 55.9, 67.9, 68.2, 116.6, 121.5, 134.1, 139.8, 195.2; UV (C_2_H_5_OH) λ_max_ = 261.4 nm, λ_max_ = 251.9 nm, λ_max_ = 243.3 nm; IR (thin film) ν_max_ 2955, 2931, 2892, 2896, 1732, 1645, 1620, 1470, 1379, 1361, 1258, 1091, 1045, 1019, 834, 734 cm^−1^; MS *m*/*z* 546 (8, M^+^).

#### 3.2.3. (*1R*,*3R*,*7E*,*22E*,*24R*)-24-Methyl-19-*nor*-20a-homo-9,10-secocholesta-5,7,22-triene-1,3,25-triol **1A**

A solution of *n*-BuLi (1.5 mL, 1.6 M, 2.4 mmol) was added under stirring to a solution of sulfone **6A** (0.8 g, 2.2 mmol) in 2.0 mL of THF at −60 °C. After 10 min, a solution of aldehyde **5** (1.5 g, 2.6 mmol) in THF (3.0 mL) was added. The mixture was stirred for 30 min at −60 °C, and brine (3 mL) was added. The resulting solution was extracted with THF (3 × 10 mL). The combined organic layer was dried with Na_2_SO_4_ (2 g) and filtered. The solvent was evaporated to obtain the crude product **7A** (ca. 2 g) as a yellow oil. Powdered Na_2_HPO_4_ (100 mg) and 10 mL of a saturated solution of Na_2_HPO_4_ in methanol were added under argon to a mixture of crude hydroxysulfones **7A** (ca. 2 g) in THF (1.0 mL), followed by sodium amalgam (20%, ca. 2 g). The mixture was stirred vigorously under argon at RT for 2 h and extracted with *t*-butyl methyl ether (3 × 10 mL). The combined organic extracts were evaporated to obtain the crude product **8A** (1.3 g) as a yellow oil. A solution of tetrabutylammonium fluoride (5.2 mL, 1 M, 5.2 mmol) was added to a solution of crude **8A** (1.3 g) in THF (10 mL). The mixture was stirred under argon at 60 °C for 1.5 h. THF extraction, silica gel filtration, preparative HPLC (Knauer RP-18 column, 8 μm, Merck, H_2_O/CH_3_CN, 40/60 (*v*/*v*)), and crystallization from CH_3_CN resulted in the formation of the crystalline triol **1A** (0.5 g) which showed a single peak on the HPLC chromatogram. ^1^H-NMR (CDCl_3_) δ 0.55 (3H, s, 18-CH_3_), 0.93 (3H, d, *J* = 6.7 Hz, 28-CH_3_), 1.01 (3H, d, *J* = 6.9 Hz, 21-CH_3_), 1.14 and 1.18 (6H, 2 × s, 27-CH_3_ and 28-CH_3_), 4.03 (1H, m, 3-H), 4.11 (1H, m, 1-H), 5.36 (1H, m, 23-H), 5.48 (1H, m, 22a-H), 5.85 (1H, d, *J* = 11.1 Hz, 7-H), 6.30 (1H, d, *J* = 11.1 Hz, 6-H); ^13^C-NMR (CDCl_3_) δ 12.1, 15.8, 18.9, 22.3, 23.4, 26.4, 26.9, 27.6, 28.9, 36.3, 37.1, 39.3, 40.4, 42.2, 44.6, 45.7, 48.4, 56.1, 56.2, 67.2, 67.4, 72.3, 115.3, 123.8, 130.7, 131.1, 133.4 142.9; UV (C_2_H_5_OH) λ_max_ = 261.2 nm, λ_max_ = 251.8 nm, λ_max_ = 243.4 nm; IR (pellet KBr) ν_max_ 3396, 2949, 2923, 2874, 2844, 1618, 1457, 1434, 1373, 1219, 1053, 1034, 976, 943, 869, 807 cm^−1^; HRMS (EI): calc. for C_28_H_46_0_3_ [M^+^]: calc. 430.3447, found 430.3445.

#### 3.2.4. (*1R*,*3R*,*7E*,*22E*,*24S*)-24-Methyl-19-*nor*-20a-homo-9,10-secocholesta-5,7,22-triene-1,3,25-triol **1B**

A solution of *n*-BuLi (1.4 mL, 1.6 M, 2.2 mmol) was added under stirring to a solution of sulfone **6B** (0.7 g, 1.9 mmol) in 2.0 mL of THF at −60 °C. After 10 min, a solution of aldehyde **5** (1.4 g, 2.4 mmol) in THF (3.0 mL) was added. The mixture was stirred for 30 min at −60 °C and brine (3 mL) was added, and then the solution was extracted with THF (3 × 10 mL). The combined organic extracts were dried with Na_2_SO_4_ (2 g) and filtered. The solvent was evaporated to obtain the crude product **7B** (ca. 1.5 g) as a yellow oil. Powdered Na_2_HPO_4_ (100 mg) and 10 mL of a saturated solution of Na_2_HPO_4_ in methanol were added under argon to a mixture of crude hydroxysulfones **7B** (ca. 1.5 g) in THF (1.0 mL), followed by sodium amalgam (20%, ca. 2 g). The mixture was stirred vigorously under argon at RT for 2 h and extracted with *t*-butyl methyl ether (3 × 10 mL). The combined organic extracts were evaporated to obtain the crude product **8B** (0.8 g) as a yellow oil. A solution of tetrabutylammonium fluoride (3.6 mL, 1 M, 3.6 mmol) was added to a solution of crude **8B** (0.8 g) in THF (10 mL). The mixture was stirred under argon at 60 °C for 1.5 h. THF extraction, silica gel filtration, preparative HPLC (Knauer RP-18 column, 8 μm, Merck, H_2_O/CH_3_CN, 40/60 (*v*/*v*)), and crystallization from CH_3_CN resulted in the formation of a crystalline triol **1B** (0.3 g) which showed a single peak on the HPLC chromatogram. ^1^H-NMR (CDCl_3_) δ 0.55 (3H, s, 18-CH_3_), 0.92 (3H, d, *J* = 6.7 Hz, 28-CH_3_), 1.02 (3H, d, *J* = 6.9 Hz, 21-CH_3_), 1.14 and 1.18 (6H, 2 × s, 27-CH_3_ and 28-CH_3_), 4.05 (1H, m, 3-H), 4.12 (1H, m, 1-H), 5.36 (1H, m, 23-H), 5.48 (1H, m, 22a-H), 5.85 (1H, d, *J* = 11.1 Hz, 7-H), 6.31 (1H, d, *J* = 11.1 Hz, 6-H); ^13^C-NMR (CDCl_3_) δ 12.1, 15.8, 18.9, 22.3, 23.5, 26.2, 27.0, 27.6, 28.9, 36.3, 37.2, 39.2, 40.4, 42.2, 44.7, 45.8, 48.5, 56.1, 56.2, 67.2, 67.4, 72.3, 115.3, 123.8, 130.7, 131.1, 133.4, 143.0; UV (C_2_H_5_OH) λ_max_ = 261.2 nm, λ_max_ = 251.8 nm, λ_max_ = 243.6 nm; IR (pellet KBr) ν_max_ 3397, 2950, 2926, 2874, 2826, 1616, 1456, 1434, 1376, 1208, 1055, 1034, 973, 944, 859, 809, 735 cm^−1^; HRMS (EI): calc. for C_28_H_46_0_3_ [M^+^]: calc. 430.3447, found 430.3437.

### 3.3. Metabolic Resistance of VDDs to CYP24A1-Dependent Degradation

The CYP24A1-dependent degradation of VDDs was examined using the recombinant human CYP24A1 (*h*CYP24A1), as described in the previous studies [19,20]. Briefly, the reaction mixture containing 2.0 μM bovine adrenodoxin, 0.2 μM bovine adrenodoxin reductase, 20 nM *h*CYP24A1, 5 µM VDD, 1 mM NADPH, 100 mM Tris-HCl (pH 7.4) and 1 mM ethylenediaminetetraacetic acid (EDTA) in a total volume of 100 µL was incubated at 37 °C, for 15 min. The metabolites were extracted with 4 volumes of chloroform/methanol (3:1) and analyzed by reversed-phase HPLC under the same conditions followed in our previous study [14]. The amount of a VDD or its metabolites was calculated from the peak area observed in the HPLC chromatogram. The percentage of metabolic conversion of a VDD was estimated as the ratio of the peak area of metabolites to the sum of the peak areas of the remaining VDD and its metabolite or metabolites (assumed as 100%).

### 3.4. Antiproliferative Activity of VDDs

To evaluate the antiproliferative activity of VDDs, either studied alone or in combination with 5-FU, two human CRC cell lines were used: HT-29 and HCT116. The cell lines were purchased from the American Type Culture Collection (ATCC, Rockville, MD, USA) in 2013 and 2017, respectively. Furthermore, HT-29 cells were positively authenticated by ATCC using the STR profiling method in 2018. These cells were cultured in a 1:1 (*v*/*v*) mixture of RPMI-1640 and Opti-MEM medium (both from Hirszfeld Institute of Immunology and Experimental Therapy, Polish Academy of Sciences (HIIET PAS), Wroclaw, Poland) supplemented with 2 mM L-glutamine, 1 mM sodium pyruvate (all from Sigma-Aldrich, Poznan, Poland), and 5% (*v*/*v*) fetal bovine serum (FBS) (HyClone, GE Healthcare, Little Chalfont, UK). HCT116 cells were cultured in McCoy’s 5A medium (Gibco, Scotland, UK) supplemented with 10% (*v*/*v*) FBS (HyClone, GE Healthcare, Little Chalfont, UK). All culture media were supplemented with the following antibiotics: streptomycin (100 μg/mL; Sigma-Aldrich, Poznan, Poland) and penicillin (100 U/mL; Polfa Tarchomin, Warsaw, Poland). The cell lines were cultured under a humid atmosphere at 37 °C with 5% CO_2_ and passaged twice a week using trypsin–EDTA solution (HIIET PAS, Wroclaw, Poland) as a detachment agent. Then, the cells were seeded at a density of 2 × 10^3^/well on 96-well plates (Sarstedt, Nümbrecht, Germany) containing 0.1 mL culture medium in each well. After 24 h of incubation, the cells were exposed for 120 h (5 days) to VDDs and 1,25D3 at the concentrations of 1000, 100, 10 and 1 nM, or only 10 nM in the case of studied VDD or 1,25D3 combined with 5-FU, while 5-FU was applied at the concentrations of 1, 0.1, 0.01 and 0.001 µg/mL (final volume in each well was 0.2 mL). The activity of PRI-5105 and PRI-5106 against CRC cells was compared with that of 1,25D3. Ethanol 99.8% used as a solvent for VDDs was diluted with the culture medium in the same manner as the studied compounds to reach the percentage concentrations of 1, 0.1, 0.01 and 0.001% (*v*/*v*) corresponding to its content at the tested concentrations of VDDs. It was observed that ethanol did not affect cell proliferation at any of the tested percentage concentrations used in the VDD solutions. After incubation, MTT assay was performed as described previously [17]. The results were calculated as the percentage of cell proliferation inhibition and then as the value of IC_50_: the concentration of the tested compound that inhibits the proliferation of cancer cell population by 50%. The percentages of cell proliferation inhibition and IC_50_ values were calculated for each experiment separately using the Prolab-3 system based on the Cheburator software described by Nevozhay [21]. The combination effect of 5-FU applied along with VDD was analyzed as described previously [22] according to the method of Chou and Talalay [23,24]. The interaction between two compounds was evaluated based on the combination index (CI) calculated for the IC_50_ of each experiment separately:

CI_A+B_ = (C_A/A+B_/C_A_) + (C_B/A+B_/C_B_) + (C_A/A+B_ × C_B/A+B_)/C_A_C_B_

where CI_A+B_ is the CI for the experimentally achieved effect of the combination of compound A (5-FU) and compound B (VDD); C_A/A+B_ is the concentration of compound A in the combination A + B; C_B/A+B_ is the concentration of compound B in the combination A + B; C_A_ is the concentration of compound A alone; and C_B_ is the concentration of compound B alone. The CI values indicated the following: <0.8, synergism; 0.8–1.2, additive effect; and >1.2, antagonism.

### 3.5. Protein Expression

Basal protein expression was analyzed in HT-29 and HCT116 cell lysates using the western blot method. The lysates were prepared using RIPA buffer with protease inhibitor cocktail (both from Sigma-Aldrich, Poznan, Poland). Total protein concentration in the cell lysates was determined using the modified Lowry method (Bio-Rad, Warsaw, Poland) according to the manufacturer’s protocol. Samples containing 50 µg of proteins were denatured with 4X Laemmli Sample Buffer (Bio-Rad, Warsaw, Poland) supplemented with β-mercaptoethanol (Sigma-Aldrich, Poznan, Poland) for 5 min at 95°C and then separated in 7.5% (E-cadherin, N-cadherin, CYP27B1, CYP24A1, 1,25D3-MARRS) or 10% (VDR) sodium dodecyl sulfate-polyacrylamide gels. After electrophoresis, the proteins were transferred from the gels to polyvinylidene difluoride membranes (0.45 µm; Merck Millipore, Burlington, MA, USA). After blocking in 5% nonfat dry milk with 0.1% phosphate-buffered saline (PBS)/Tween-20 (PBST) (PBS from HIIET PAS, Wroclaw, Poland; Tween-20 from Sigma-Aldrich, Poznan, Poland) at RT for 1 h, the membranes were washed thrice for 10 min with 0.1% PBST and then incubated overnight at 4 °C with the following primary rabbit polyclonal antibodies: anti-VDR, anti-1,25D3-MARRS, anti-E-cadherin, anti-N-cadherin, anti-CYP27B1 and anti-CYP24A1 (all from Santa Cruz Biotechnology, Santa Cruz, CA, USA). After incubation with a primary antibody, the membranes were washed thrice for 10 min with 0.1% PBST and then incubated at RT for 1 h with horseradish peroxidase (HRP)-conjugated secondary antirabbit antibody. After incubation, the membranes were washed thrice for 5 min with 0.1% PBST, and the protein bands were detected using an ECL system. Then, to determine the expression of β-actin, the same membranes (for each tested protein) were incubated with mouse anti-β-actin-HRP monoclonal antibody (Santa Cruz Biotechnology, Santa Cruz, CA, USA) at RT for 1 h, washed thrice for 10 min with 0.1% PBST, and used for the ECL detection. Chemiluminescence was visualized using an Image Station 4000 system (Carestream Health, Rochester, NY, USA). The results were normalized to β-actin used as the internal loading control. Densitometric analysis was performed using ImageJ 1.48v software (National Institutes of Health, Bethesda, MA, USA).

### 3.6. Statistical Analysis

Statistical analysis was performed using GraphPad Prism 7 (GraphPad Software Inc., San Diego, CA, USA). The assumptions of analysis of variance (ANOVA) were checked using Shapiro–Wilk normality test and Brown–Forsythe test. If the assumptions of the parametric test were found to be fulfilled, one- or two-way ANOVA followed by Dunnett’s or Sidak’s multiple comparison test was run. The specific tests used for data analysis are listed in the figure legends. Differences with a *p*-value of less than 0.05 were considered statistically significant.

## 4. Conclusions

The study revealed that the absolute configuration at C-24 in the 19-*nor* analogs having an extended vitamin D_2_-like side chain did not significantly influence their biological activity, as both PRI-5105 and PRI-5106 showed not only similar antiproliferative activity but also comparable resistance against the recombinant human CYP24A1. We also found that neither the 19-*nor* modification of the A-ring nor the side-chain extension by a single carbon unit increased the resistance of the analogs to the CYP24A1-dependent metabolism. Similar to our PRI-1906 and PRI-1907, it is the very specific conjugation of the two double bonds at C-22 and C-24 in the side chain that determines the resistance of the VDDs of 1,25D2 to the CYP24A1-dependent catabolism. Additionally, both analogs revealed a similar antiproliferative activity against HT-29 CRC cells but higher than 1,25D3. Furthermore, PRI-5105 and PRI-5106 significantly enhanced the cell growth-inhibitory activity of 5-FU on HT-29 cell line. However, this effect was observed only in the case of HT-29 cells but not HCT116 cells. HT-29 cells exhibited a higher basal level of VDR and E-cadherin as well as an enhanced ratio of CYP27B1 to CYP24A1 compared to HCT116 cells, which might contribute to their better response to the activity of VDDs compared to HCT116 cells. However, further investigation is required to identify the molecular factors underlying the differences in the response of CRC cells to VDDs. In conclusion, although PRI-5105 and PRI-5106 showed a relatively high anticancer potential, they exhibited undesired high metabolic conversion.

## Figures and Tables

**Table 1:** Human CYP24A1-dependent metabolic conversion of the 19*-nor* VDDs of 1,25D2 (PRI-5105 and PRI-5106) in comparison with their most active metabolite of vitamin D_2_ (1,25D2).

Compound	1,25D2	PRI-5105	PRI-5106
Metabolic conversion (%)	33 ± 4.2	25 ± 1.9	26 ± 3.3

Data represent the means ± SD of at least three independent experiments.

**Table 2:** IC_50_ and combination index (CI) values of 5-FU used alone or together with VDDs against human HT-29 and HCT116 colorectal cancer (CRC) cell lines.

Compound/CI	IC_50_ [µM]	
	HT-29	HCT116
1,25D3	0.34 ± 0.4	nd
PRI-5105	0.15 ± 0.1	nd
PRI-5106	0.09 ± 0.01	nd
5-FU	2.09 ± 0.5	2.60 ± 0.1
5-FU + 1,25D3	1.49 ± 0.7	3.07 ± 0.5
CI	0.8 ± 0.3	-
5-FU + PRI-5105	0.71 ± 0.4 **	2.66 ± 0.6
CI	0.4 ± 0.2	-
5-FU + PRI-5106	0.78 ± 0.3 **	2.69 ± 0.4
CI	0.5 ± 0.04	-

Data represent the mean ± SD of four independent experiments; nd represents that values were not determined in the concentration range used. Because of poor sensitivity of HCT116 cells to the proliferation-inhibiting effect of the studied analogs the calculation of CI in the case of this cell line was impossible. Statistical analysis was carried out using parametric one-way ANOVA followed by Dunnett’s multiple comparison test (** *p* ≤ 0.01 as compared to 5-FU).

## Supplementary Materials

The following are available online at https://www.mdpi.com/1422-0067/21/2/642/s1, Figure S1: HPLC profiles of VDDs, PRI-5105 and PRI-5106, before and after incubation with *h*CYP24A1 Figure S2: Chemical structures of VDDs of 1,25D2, Figure S3: The ratio of the EMT markers (E- to N-cadherin) and the enzymes that catalyze the hydroxylation of vitamin D metabolites into its active or inactive form (CYP27B1 to CYP24A1, respectively) in HT-29 and HCT116 CRC cell lines.

## Abbreviations

1,25D2	1,25-dihydroxyvitamin D_2_
1,25D3	1,25-dihydroxyvitamin D_3_
5-FU	5-fluorouracil
CMS	consensus molecular subtypes
CRC	colorectal cancer
CYP24A1	25-hydroxyvitamin D_3_-24-hydroxylase
CYP27B1	25-hydroxyvitamin D_3_-1α-hydroxylase
EMT	epithelial-mesenchymal transition
MARRS	membrane associated rapid response steroid-binding protein
VDD	vitamin D derivative
VDR	vitamin D receptor
