# Supplementary material for: Synthesis, CYP24A1-Dependent Metabolism and Antiproliferative Potential against Colorectal Cancer Cells of 1,25-Dihydroxyvitamin D2 Derivatives Modified at the Side Chain and the A-Ring
Source: Int J Mol Sci. 2020 Jan 18;21(2):642. doi: 10.3390/ijms21020642 (PMC7013581; doi:10.3390/ijms21020642)
Supplement: Supplementary file 1 [file ijms-21-00642-s001.pdf]

International Journal of *Molecular Sciences*

MDPI

Supplementary Materials

Synthesis, CYP24A1-Dependent Metabolism and Antiproliferative Potential against Colorectal Cancer Cells of 1,25-dihydroxyvitamin D2 Derivatives Modified in the Side-Chain and in the A-Ring

Magdalena Milczarek[1,*], Michał Chodyński[2], Anita Pietraszek[2], Martyna Stachowicz-Suhs[1], Kaori Yasuda[3], Toshiyuki Sakaki[3], Joanna Wietrzyk[1] and Andrzej Kutner[4]

[1] Hirszfeld Institute of Immunology and Experimental Therapy, Polish Academy of Sciences, 12 Rudolfa Weigla, 53-114 Wrocław, Poland; magdalena.milczarek@hirszfeld.pl, martyna.stachowicz@hirszfeld.pl, joanna.wietrzyk@hirszfeld.pl

[2] Chemistry Department, Pharmaceutical Research Institute, 8 Rydygiera, 01-793 Warsaw, Poland; m.chodynski@ifarm.eu, a.pietraszek@ifarm.eu

[3] Department of Biotechnology, Faculty of Engineering, Toyama Prefectural University, Imizu, Toyama, Japan 939-0398, kyasuda@pu-toyama.ac.jp, tsakaki@pu-toyama.ac.jp

[4] Department of Bioanalysis and Drug Analysis, Faculty of Pharmacy with the Laboratory Medicine Division, Medical University of Warsaw, 1 Banacha, 02-097 Warsaw, Poland; akutner@chem.uw.edu.pl

* Correspondence: magdalena.milczarek@hirszfeld.pl; Tel.: +48-71-337-1172, ext. 171 (M.M.)

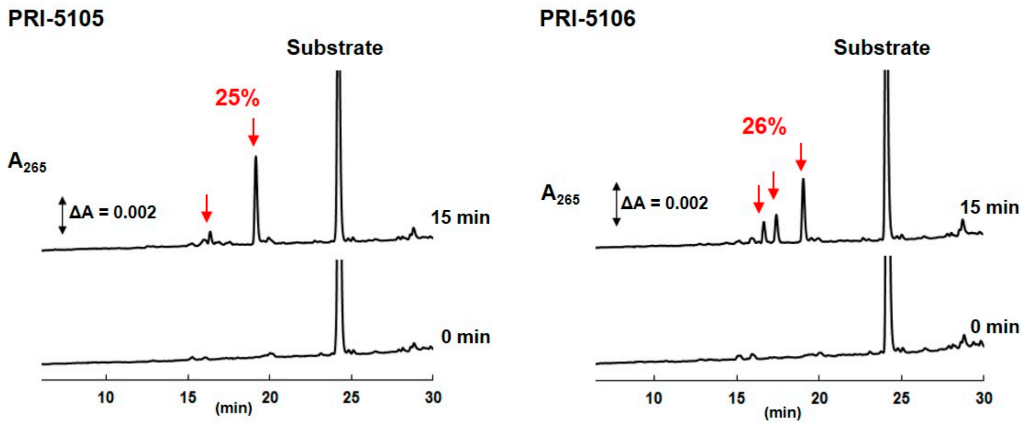

Figure S1. HPLC profiles of VDDs, PRI-5105 and PRI-5106, before and after incubation with *h*CYP24A1. The peaks marked with arrows indicate putative metabolites. ΔA indicates the absorbance difference at 265 nm. The upper chromatograms represent reaction mixture profiles following incubation with *h*CYP24A1 for 15 min. The lower chromatograms were obtained at the starting point.

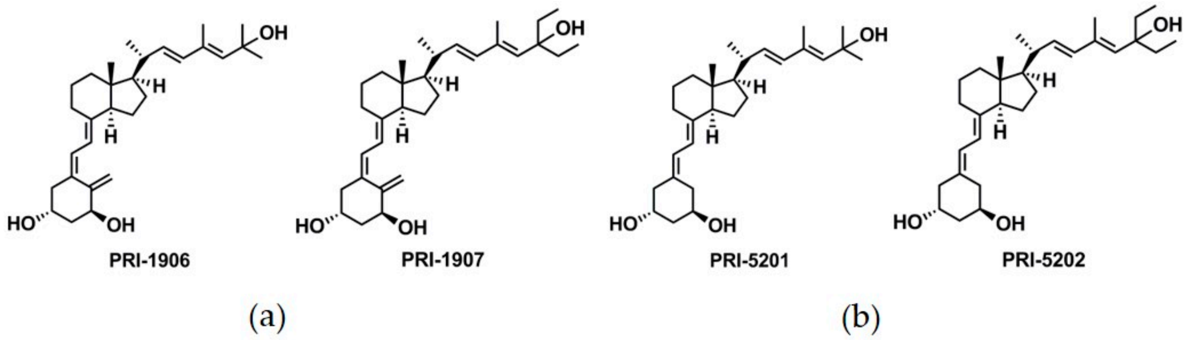

Figure S2. Chemical structures of VDDs of 1,25D2. (a) Single-point modified VDDs: PRI-1906, PRI-1907 (side-chain modified VDDs of 1,25D2); (b) Double-point modified VDDs: PRI-5201 and PRI-5202 (19-*nor* modification of PRI-1906 and PRI-1907, respectively).

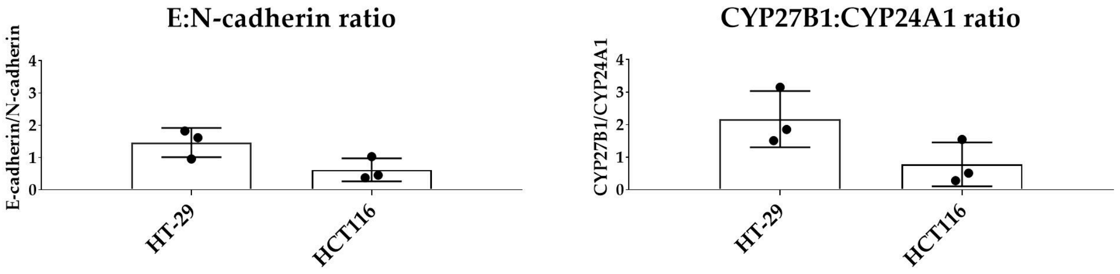

Figure S3. The ratio of the EMT markers (E- to N-cadherin) and the enzymes that catalyze the hydroxylation of vitamin D metabolites into its active or inactive form (CYP27B1 to CYP24A1, respectively) in HT-29 and HCT116 CRC cell lines. The basal expression of proteins was evaluated by western blot, then densitometric analysis of bands of protein of interest as a ratio to β-actin was performed. Data presented as mean with SD and with data of individual samples for 3 independent experiments.
